# The HyVac4 Subunit Vaccine Efficiently Boosts BCG-Primed Anti-Mycobacterial Protective Immunity

**DOI:** 10.1371/journal.pone.0039909

**Published:** 2012-06-29

**Authors:** Rolf Billeskov, Tara T. Elvang, Peter L. Andersen, Jes Dietrich

**Affiliations:** Department of Infectious Disease Immunology, Statens Serum Institut, Copenhagen, Denmark; University of Delhi, India

## Abstract

**Background:**

The current vaccine against tuberculosis (TB), BCG, has failed to control TB worldwide and the protective efficacy is moreover limited to 10–15 years. A vaccine that could efficiently boost a BCG-induced immune response and thus prolong protective immunity would therefore have a significant impact on the global TB-burden.

**Methods/Findings:**

In the present study we show that the fusion protein HyVac4 (H4), consisting of the mycobacterial antigens Ag85B and TB10.4, given in the adjuvant IC31® or DDA/MPL effectively boosted and prolonged immunity induced by BCG, leading to improved protection against infection with virulent *M. tuberculosis* (M.tb). Increased protection correlated with an increased percentage of TB10.4 specific IFNγ/TNFα/IL-2 or TNFα/IL-2 producing CD4 T cells at the site of infection. Moreover, this vaccine strategy did not compromise the use of ESAT-6 as an accurate correlate of disease development/vaccine efficacy. Indeed both CD4 and CD8 ESAT-6 specific T cells showed significant correlation with bacterial levels.

**Conclusions/Significance:**

H4-IC31® can efficiently boost BCG-primed immunity leading to an increased protective anti-M.tb immune response dominated by IFNγ/TNFα/IL-2 or TNFα/IL2 producing CD4 T cells. H4 in the CD4 T cell inducing adjuvant IC31® is presently in clinical trials.

## Introduction

Tuberculosis (TB) is a chronic respiratory disease caused by infection with the intracellular pathogen *Mycobacterium tuberculosis* (M.tb) which kills almost two million people each year [1]. Infection with M.tb leads to a quiescent latent infection rather than active disease in more than 90% of infected individuals and one third of the world’s population is estimated to harbour a latent TB infection [2], [3]. The current vaccine, Bacillus Calmette-Guérin (BCG), is an attenuated strain of the closely related *Mycobacterium bovis.* It protects children against severe disseminated forms of TB. However, protection against pulmonary disease varies between 0–80% and is generally lowest in endemic areas [4]. Due to the protective efficacy against paediatric forms of disseminated TB and the worldwide coverage through the WHO Extended Program of Immunization (EPI), BCG is not likely to be replaced within the near future.

BCG has been shown to induce a T helper cell 1 (Th1) biased immune response and it has been suggested that gradual exhaustion of an insufficient pool of central memory T cells induced by BCG may be responsible for the declining protection against TB, which is estimated to last for only 10–15 years [5], [6], [7]. Several controlled trials have been performed to evaluate the effect of revaccinating with BCG in order to boost BCG immunity and T cell memory. However, these trials demonstrated that revaccination with BCG did not improve protection [8], [9], probably because BCG induced immunity from the first dose inhibits the response of the second BCG-dose. In line with this, continuous exposure to environmental mycobacteria has been suggested as an important factor leading to the limited protection of BCG [4], [10], [11]. Although still debated in the field, animal studies have shown that exposure to environmental mycobacteria did in fact inhibit survival of BCG after vaccination, leading to decreased protection [12]. Importantly, the protection of adjuvanted subunit vaccines is not affected by environmental mycobacteria exposure. Thus, environmental mycobacteria could be a major factor responsible for the limited efficacy of BCG and accordingly development of an improved vaccine to boost the partial protective immune response in BCG vaccinated individuals is of high priority in the effort to control TB.

In the search for new and improved vaccines against TB much attention has focused on secreted mycobacterial proteins. These proteins were indeed shown to be protective in animals and also recognized in infected humans [13], [14], [15]. Our laboratory has developed a subunit vaccine named Hybrid1 (H1) consisting of two fused protein antigens, Ag85B and ESAT-6, both present in short term culture filtrate and recognized in infected individuals. H1 is protective in several animal models [16], [17] and produced promising results in a clinical phase I trial [18] when formulated in the IC31® adjuvant. However, as ESAT-6 is an extremely valuable diagnostic tool incorporated into several commercial diagnostic kits [19], [20], and H1 immunization may interfere with TB-diagnosis, we therefore replaced ESAT-6 with TB10.4 to generate the vaccine H4 (Ag85B-TB10.4) [21]. TB10.4 was chosen as this protein is highly expressed by BCG and M.tb and strongly recognized in BCG vaccinated and/or infected humans. H4 on its own has been showed to induce efficient anti-TB protection in mice when given in IC31® or DDA/MPL adjuvants [21], [22].

In the present study we evaluated for the first time in the murine TB model the ability of the H4-vaccine in IC31® (or DDA/MPL) to boost and prolong the immune response induced by BCG. We found that H4 efficiently boosted effector and memory CD4 T cells primed by BCG, leading to a stronger and prolonged immune response dominated by multifunctional T cells. This in turn improved the protection against infection with M.tb. Finally we also observed that ESAT-6 specific CD4 as well as CD8 T cells correlated inversely with protection against M.tb. The implications of our findings for the development of novel anti TB vaccines are discussed.

## Results

### Boosting BCG with H4/IC31 Leads to Enhanced and Prolonged Immune Responses

**Figure 1:**
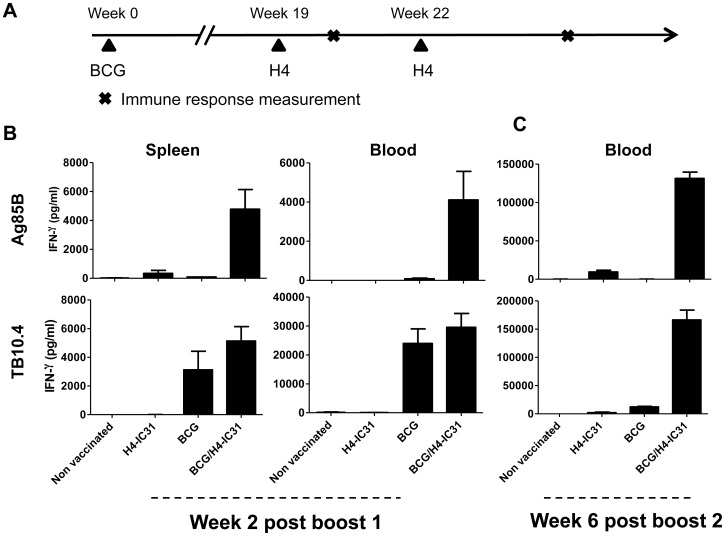
H4-IC31® boost BCG-primed immune responses and memory. Mice that received BCG were immunized at week 0 and the H4-IC31® and BCG/H4-IC31® groups were subsequently immunized with H4 in IC31® at 19 and 22 weeks after initiating the experiment (A). The amount of IFNγ produced by splenocytes and PBMCs (pooled from five mice per group) in response to Ag85B and TB10.4 stimulation was evaluated by ELISA. The responses were evaluated two weeks after the first H4-IC31® immunization (B), and memory responses were evaluated six weeks after the second immunization in PBMCs pooled from n = 15 mice per group (C). Bars represent means and standard deviations of triplicate 72 hour culture-supernatants. Stimulation with media alone as well as ESAT-6 as a negative control antigen resulted in IFNγ secretion below 300 pg/ml (data not shown). This experiment was repeated once with similar results.

We first analysed the ability of H4 given in IC31® to boost BCG-primed immunity in mice. Mice were immunized as shown in figure 1A. Two weeks after the first boost with H4-IC31, mice were sacrificed and the amount of IFNγ produced after *in vitro* stimulation with each of the vaccine antigens was assessed by ELISA (Fig. 1B). The results showed that the level of IFNγ produced in the BCG/H4-IC31® boost group after Ag85B-stimulation of PBMCs (4109±1458 pg/ml) and splenocytes (4777±1360 pg/ml) was strongly increased compared to the remaining groups, which produced less than 350 pg/ml IFNγ (Fig. 1B, upper panel). Regarding the TB10.4 response both BCG and BCG/H4-IC31® vaccinated mice produced strong IFNγ responses (Fig. 1B, lower panel). We next evaluated the vaccine-induced memory response in mice six weeks after the second booster immunization with H4-IC31®. The results demonstrated that the BCG/H4-IC31® group exhibited an increased response compared to the other vaccine groups (Fig. 1C). Stimulating PBMC’s in vitro with Ag85B led to production of 131453±8236 pg/ml IFNγ in the BCG/H4-IC31® group, compared to less than 100 pg/ml in the non-vaccinated or BCG-vaccinated groups. In mice vaccinated only with H4-IC31 we observed 9379±2319 pg/ml IFNγ. In line with this, TB10.4 stimulation showed a strong increase in the production of IFNγ in the BCG/H4-IC31® group (158979±21338 pg/ml), compared to the non-vaccinated (26±12 pg/ml), H4-IC31® (2043±1107 pg/ml) and BCG (12222±1020 pg/ml) vaccinated mice (Fig. 1C). Thus, two booster vaccinations with H4-IC31® induced a stronger and prolonged response against both the vaccine antigens.

### Boosting BCG with H4-IC31® Induces Polyfunctional CD4 T Cells with a Memory and Effector Phenotype

**Figure 2:**
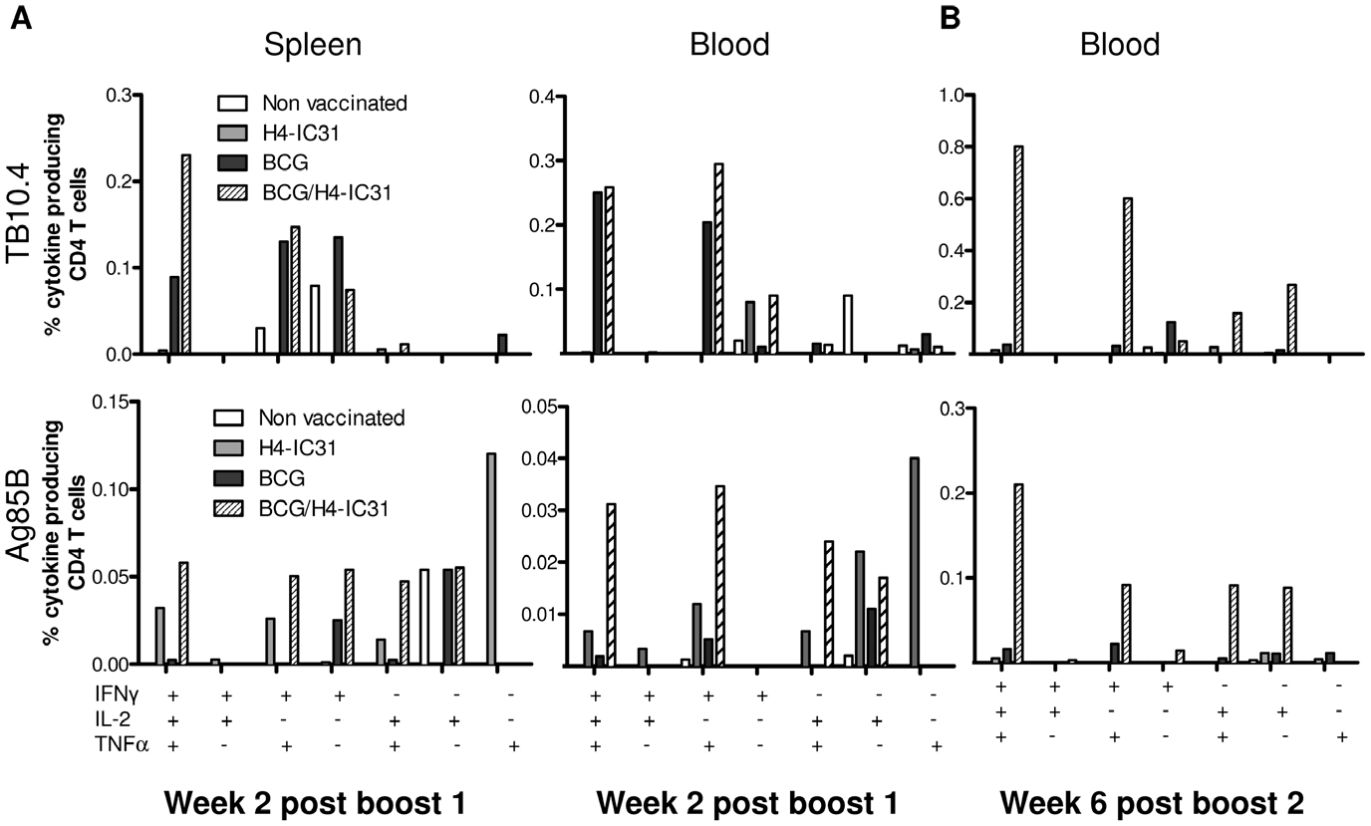
Boosting BCG with H4-IC31® induces polyfunctional memory and effector CD4 T cells. Mice were immunized as depicted in figure 1A, and parallel with the ELISA analysis, splenocytes and PBMCs (pooled from n = five mice/group) obtained two weeks after the first H4-IC31® immunization (A) and PBMCs (pooled from n = 15 mice/group) obtained six weeks after the second immunization (B) were used for intracellular cytokine analysis by flow cytometry. Cells were stimulated with TB10.4 (upper panels) and Ag85B (lower panels). Bars represent the proportions of CD4 T cell subsets producing different cytokines as indicated on the X-axis. Background levels obtained in media-stimulated samples has been subtracted. This experiment was repeated once with similar results.

We next evaluated the T cell phenotype induced by the different vaccination strategies. Two weeks after the first H4 booster immunization, splenocytes and PBMCs were stimulated *in vitro* with TB10.4 or Ag85B and the phenotype of responding CD4 T cells was evaluated by multicolor intracellular cytokine staining (ICS) and flow cytometry. The groups immunized with BCG and BCG/H4-IC31® exhibited the strongest responses against TB10.4. Moreover, increased levels of triple-positive CD4 T cells, expressing IFNγ, TNFα and IL-2 simultaneously, were observed in the BCG/H4-IC31® group compared to the BCG alone group in the spleen (Fig. 2A and table 1). The H4-IC31® and BCG/H4-IC31® groups both showed higher Ag85B responses compared to naïve and BCG-immunized mice although the Ag85B levels were generally lower than the TB10.4 responses (Fig. 2A and table 1). A similar boosting effect was also observed in the blood 2 weeks after the first booster vaccination, although responding CD4 T cells in the blood displayed slightly more effector-like phenotypes (Fig. 2A).

**Table 1:** Percentages of CD4 T cells producing cytokines after first and second H4-IC31-boost and after infection.

Percentage of CD4 T cells specific for TB10.4								
**Time**	**Week 2 post boost 1 spleen**				**Week 2 post boost 1 blood**			
**Group**	**Non vacc.**	**H4-IC31**	**BCG**	**BCG/H4-IC31**	**Non vacc.**	**H4-IC31**	**BCG**	**BCG/H4-IC31**
	**Relative** a **(%)**	**Relative (%)**	**Relative (%)**	**Relative (%)**	**Relative** a **(%)**	**Relative (%)**	**Relative (%)**	**Relative (%)**
IFNγ^+^IL-2^+^TNFα^+^	0,00	42,84	23,67	49,74	0,00	1,93	48,93	38,72
IFNγ^+^IL-2^+^TNFα^-^	0,00	0,00	0,00	0,00	0,00	0,00	0,35	0,00
IFNγ^+^IL-2^−^TNFα^+^	27,82	0,00	34,57	31,82	0,00	0,00	39,96	44,18
IFNγ^+^IL-2^−^TNFα^-^	72,18	0,00	35,90	16,00	16,39	90,18	1,96	13,50
IFNγ^-^IL-2^+^TNFα^+^	0,00	57,16	0,00	2,44	0,00	0,00	2,94	1,95
IFNγ^-^IL-2^+^TNFα^-^	0,00	0,00	0,00	0,00	73,77	0,00	0,00	0,15
IFNγ^-^IL-2^−^TNFα^+^	0,00	0,00	5,85	0,00	9,84	7,89	5,87	1,50
Total response	100,00	100,00	100,00	100,00	100,00	100,00	100,00	100,00
**Percentage of CD4 T cells specific for Ag85B**								
	**Relative** **(%)**	**Relative** **(%)**	**Relative** **(%)**	**Relative** **(%)**	**Relative** **(%)**	**Relative** **(%)**	**Relative** **(%)**	**Relative** **(%)**
IFNγ^+^IL-2^+^TNFα^+^	0,00	16,35	2,99	21,94	0,00	7,38	10,53	29,21
IFNγ^+^IL-2^+^TNFα^-^	0,00	1,38	0,00	0,00	0,00	3,69	0,00	0,00
IFNγ^+^IL-2^−^TNFα^+^	0,00	13,28	0,00	18,96	39,02	13,20	28,49	32,42
IFNγ^+^IL-2^−^TNFα^-^	0,00	0,51	29,75	20,42	0,00	0,00	0,00	0,00
IFNγ^-^IL-2^+^TNFα^+^	0,00	7,15	2,99	17,88	0,00	7,38	0,00	22,46
IFNγ^-^IL-2^+^TNFα^-^	100,00	0,00	64,27	20,80	60,98	24,25	60,98	15,91
IFNγ^-^IL-2^−^TNFα^+^	0,00	61,32	0,00	0,00	0,00	44,09	0,00	0,00
Total response	100,00	100,00	100,00	100,00	100,00	100,00	100,00	100,00
**Percentage of CD4 T cells specific for TB10.4**								
**Time**	**Week 6 post boost 2 blood**				**Week 6 post post infection lungs**			
**Group**	**Non vacc.**	**H4-IC31**	**BCG**	**BCG/H4-IC31**	**Non vacc.**	**H4-IC31**	**BCG**	**BCG/H4-IC31**
	**Relative** **(%)**	**Relative** **(%)**	**Relative** **(%)**	**Relative** **(%)**	**Relative** **(%)**	**Relative** **(%)**	**Relative** **(%)**	**Relative** **(%)**
IFNγ^+^IL-2^+^TNFα^+^	0,00	30,05	17,97	42,48	18,63	34,90	56,60	61,62
IFNγ^+^IL-2^+^TNFα^-^	0,00	0,00	0,00	0,21	0,33	0,39	0,15	0,05
IFNγ^+^IL-2^−^TNFα^+^	0,00	0,00	15,60	31,93	65,43	39,20	25,53	20,06
IFNγ^+^IL-2^−^TNFα^-^	100,00	9,86	58,63	2,66	7,56	3,87	0,57	0,73
IFNγ^-^IL-2^+^TNFα^+^	0,00	49,49	0,00	8,50	1,15	7,65	6,44	11,18
IFNγ^-^IL-2^+^TNFα^-^	0,00	10,60	7,81	14,23	0,00	0,30	0,04	0,06
IFNγ^-^IL-2^−^TNFα^+^	0,00	0,00	0,00	0,00	6,89	13,68	10,67	6,30
Total response	100,00	100,00	100,00	100,00	100,00	100,00	100,00	100,00
**Percentage of CD4 T cells specific for Ag85B**								
	**Relative** **(%)**	**Relative** **(%)**	**Relative** **(%)**	**Relative** **(%)**	**Relative** **(%)**	**Relative** **(%)**	**Relative** **(%)**	**Relative** **(%)**
IFNγ^+^IL-2^+^TNFα^+^	0,00	26,43	24,62	42,23	22,56	37,26	42,86	44,01
IFNγ^+^IL-2^+^TNFα^-^	0,00	0,00	0,00	0,61	0,10	0,29	0,11	0,12
IFNγ^+^IL-2^−^TNFα^+^	0,00	0,00	33,85	18,35	58,96	25,71	19,99	13,69
IFNγ^+^IL-2^−^TNFα^-^	0,00	0,00	0,00	2,82	6,61	2,38	4,53	0,84
IFNγ^-^IL-2^+^TNFα^+^	0,00	0,00	8,45	18,30	2,94	13,89	9,69	23,62
IFNγ^-^IL-2^+^TNFα^-^	0,00	53,95	16,17	17,70	0,00	0,70	8,49	0,31
IFNγ^-^IL-2^−^TNFα^+^	0,00	19,62	16,92	0,00	8,82	19,77	14,33	17,41
Total response	0,00	100,00	100,00	100,00	100,00	100,00	100,00	100,00

a The relative proportion of the responding CD4 T cells producing combinations of IFNγ, TNFα and/or IL-2 as listed when stimulated with either *in vitro* TB10.4 or Ag85B, assessed by intracellular cytokine staining and flowcytometry.

In order to assess the phenotype of the T cells in later phases of the response, mice were bled six weeks after the second H4-IC31® immunization and PBMCs were stimulated *in vitro* with TB10.4 or Ag85B. The data showed that boosting BCG with H4-IC31® clearly enhanced the response against TB10.4 in the late phase (Fig. 2B). The responding CD4 T cells in the BCG/H4-IC31® group could primarily be divided into cells expressing all three cytokines (42% of responding CD4 T cells), cells expressing TNFα and IFNγ (32% responding CD4 T cells), or cells expressing TNFα and IL-2 (8.5% responding CD4 T cells; table 1). Interestingly, the TB10.4-specific CD4 T cells in the BCG group were dominated by a IFNγ^+^ terminally differentiated phenotype. This could explain the low level of memory immune response observed following BCG immunization (Fig. 2 B and table 1), as T cell phenotypes have been suggested to express a specific pattern of cytokines: 1) IFNγ^+^TNFα^+^IL-2^+^ cells representing effector memory T cells, 2) TNFα^+^IL-2^+^ cells representing central memory cells and 3) IFNγ^+^ cells representing terminally differentiated T cells [25], [26]. We will refer to these designations from here on.

The same overall pattern was seen when the cells were stimulated with Ag85B (Fig. 2B, lower panel). Thus, at week 6 after the second booster vaccination, the Ag85B response was strongest in the BCG/H4-IC31® group (Fig. 2B) and the responding CD4 T cells consisted of 44% polyfunctional IFNγ^+^TNFα^+^IL-2^+^ T cells compared to only 27% in the BCG group which in turn contained relatively more effector-like IFNγ^+^TNFα^+^ T cells compared to the BCG/H4-IC31® group (table 1).

Thus, our results show that boosting BCG with H4-IC31® could enhance and prolong both the response as well as the induction and maintenance of T cells producing multiple cytokines.

### Vaccine-induced Immune Responses after Infection with Virulent M.tb

An important feature of any vaccine against infection with M.tb is if/how the vaccine-induced immune responses are maintained in the face of an infection. To examine this we analysed the immune responses in the lungs of BCG/H4-IC31® immunized mice after an aerosol-infection with virulent M.tb. Mice were infected 7 weeks after the last H4-IC31® booster immunization and immune responses were evaluated by stimulating lung cells *in vitro* with the vaccine antigens or ESAT-6 as a marker of the infection driven response. Antigen specific T cell phenotypes were evaluated by ICS and flow cytometry six weeks after infection in order to assess to which degree the vaccine-induced T cells were recruited to the lungs and whether the infection influenced the phenotype of these cells.

**Figure 3:**
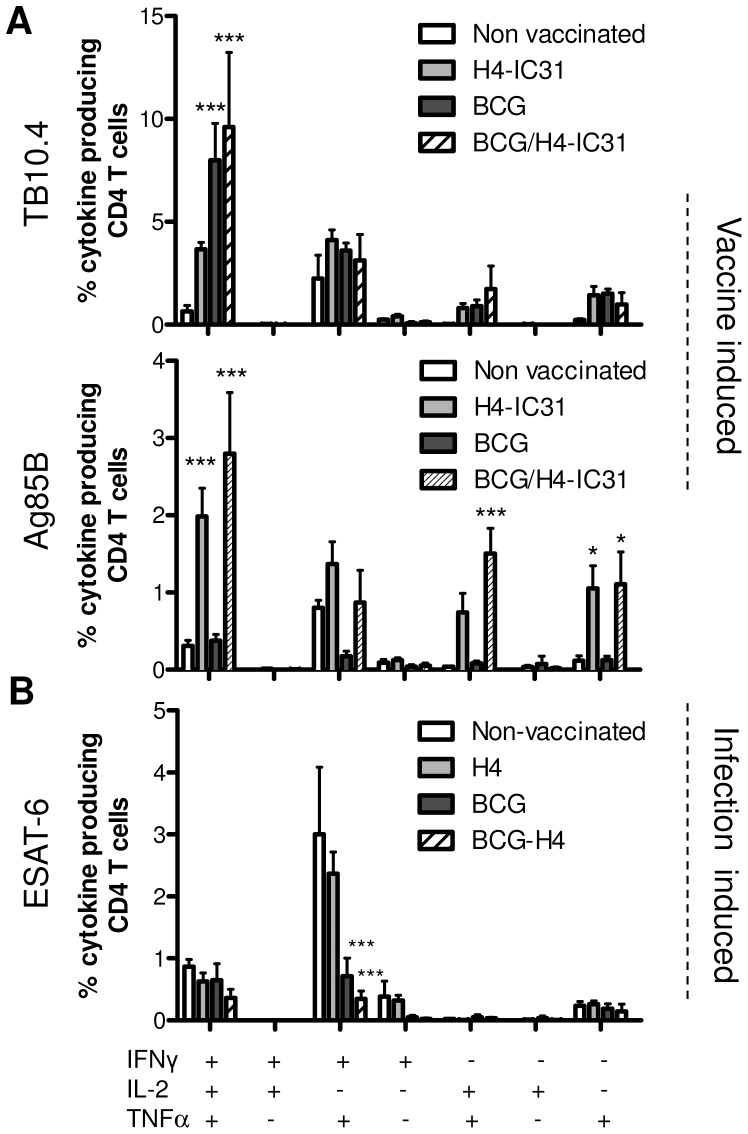
Polyfunctional vaccine specific T cells recruited to the lungs after infection. Mice immunized as described in the experiment shown in figure 1 were infected by the aerosol route with virulent M.tb Erdman seven weeks after the last H4-IC31® booster immunization. Six weeks after infection lung lymphocytes from three pools consisting of two half lungs per pool were used from each group for intracellular cytokine analysis by flow cytometry. Cells were stimulated with the indicated antigens derived from H4 (Ag85B and TB10.4; A) or ESAT-6 (B) as specified in the graph. Bars represent the proportions of lung CD4 T cell subsets producing different cytokines in response to stimulation as indicated on the X-axis. Background levels obtained in media-stimulated samples has been deducted. *, p<0.05, **, p<0.01, ***, p<0.001, proportion of cytokine producing CD4 T cells subsets compared to non-vaccinated controls using one-way ANOVA and Tukey’s post-test for multiple comparisons. This experiment was repeated once with similar results.

The results showed that after infection groups immunized with H4-IC31® showed the highest response against Ag85B, whereas groups immunized with BCG, with or without the H4 boost, exhibited the highest response against TB10.4 (Fig. 3A). This is in agreement with previous observations showing that Ag85B is the most immunogenic antigen in H4 [21] whereas BCG induce stronger responses to TB10.4 [23], [27]. Although we observed no major differences in the TB10.4 responses in the BCG and BCG/H4-IC31® groups, there was a trend towards a more polyfunctional/central memory (and less effector-like) phenotype in the BCG/H4-IC31® group (Fig. 3A, table 1). Thus, 15.6% of all lung CD4 T cells responded to TB10.4 in the BCG/H4-IC31® group and of these 61.6% were IFNγ/TNFα/IL-2 producing polyfunctional cells (compared to 56.6% in the BCG group), 20.1% were IFNγ^+^TNFα^+^ effector-like cells (compared to 25.5% in the BCG group), and 11.2% were IL-2^+^TNFα^+^ central memory-like T cells (compared to 6.4% in the BCG group) (Fig. 3A and table1). The highest proportion of IFNγ^+^TNFα^+^ and IFNγ^+^ effector-like TB10.4-specific T cells was observed in the non-vaccinated group (Fig. 3A and table 1).

In terms of Ag85B, the strongest responses were seen in the BCG/H4-IC31® (6.4% of CD4 T cells) and H4-IC31® groups (5.3% of CD4 T cells; figure 3A and table 1). In the BCG/H4-IC31® group we generally observed a higher proportion of Ag85B specific cells represented by polyfunctional triple-positive and TNFα^+^IL-2^+^ central memory-like phenotypes, and lower levels of effector phenotypes TNFα^+^IFNγ^+^ and/or IFNγ^+^ compared to the remaining groups (Fig 3A and table 1).

The same overall pattern of responses was also observed in the spleens of mice (data not shown). Interestingly, in contrast to the response against the vaccine antigens, the response against ESAT-6 was dominated by IFNγ^+^TNFα^+^ effector T cells, and was highest in groups that exhibited the lowest responses against TB10.4 (Fig. 3B). This is most probably because these cells were induced by the infection itself, which has been shown to induce T cells of that phenotype [28].

Thus, the BCG/H4-IC31® group showed the highest ability to induce and maintain a strong response against both of the H4-contained vaccine antigens during an infection. Furthermore, this response against the vaccine antigens was in particular dominated by T cells with a polyfunctional/central memory-like phenotype that were recruited to the site of infection.

### Boosting BCG with H4-IC31® Improves the Protective Efficacy

**Figure 4:**
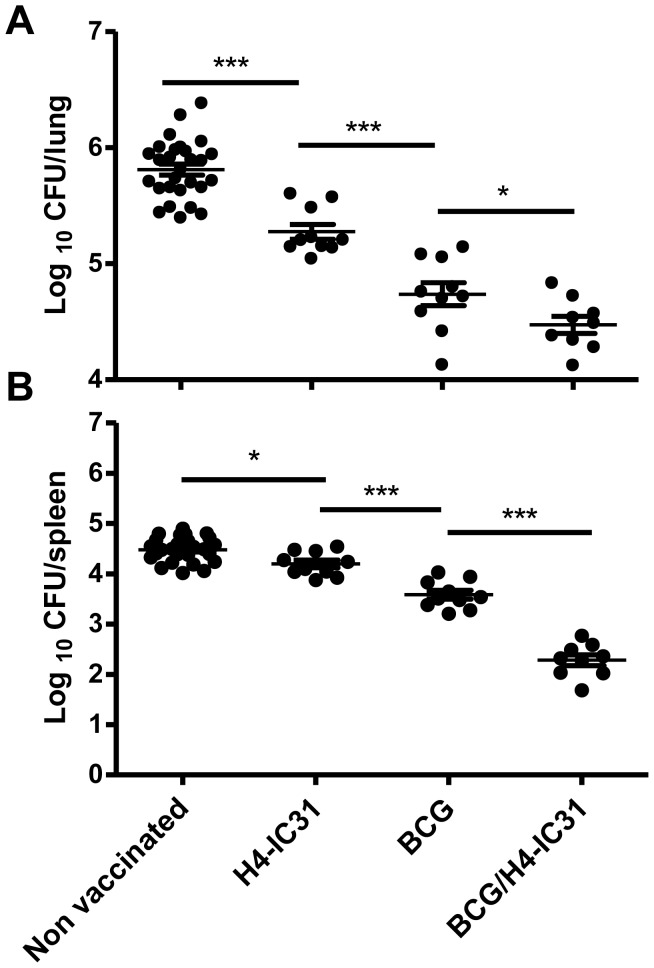
Boosting BCG with H4-IC31® improves BCG-derived protection. Six weeks after infection the level of protection was assessed in the lungs (upper panel) and spleens (lower panel) of infected mice. Each data-point represents the log_10_ CFU value of individual mice (N = 29 for non-vaccinated controls and n = 9−10 for the vaccination groups). Means and SEM for each group is indicated. *, p<0.05, **, p<0.01, ***, p<0.001, using one-way ANOVA and Newman-Keuls post test for multiple comparisons.

We next examined the level of protection against an M.tb-challenge offered by boosting BCG with H4-IC31® compared to BCG-immunization alone. The number of bacteria in the lungs and spleens were enumerated six weeks after infection. The results showed that boosting BCG with H4-IC31® significantly reduced CFU in the lung and spleen compared to BCG alone (Fig. 4 and table S1). Optimal protection with H4 formulated in IC31® has previously been demonstrated with three immunizations [21], however, even with a suboptimal vaccination scheme only involving two immunizations, H4-IC31® protected mice significantly against M.tb-challenge (Fig. 4). Moreover, in three different experiments, a similar level of protection was observed when boosting BCG with H4 in IC31® or DDA/MPL ranging from 1.2–1.5 log_10_ reduction in CFU in the lungs compared to non-vaccinated mice. Importantly, we only observed a significant improvement in protection compared to BCG when BCG itself conferred a reduction of one log_10_ CFU or less (Fig. S1). The lower level of protection obtained from BCG in Experiment 1 in figure S1 is most likely related to the prolonged duration between BCG and infection in this experiment (see materials and methods for details).

### Monitoring Vaccine-efficacy Through Antigen Specific CD4 or CD8 Responses after Infection

ESAT-6 is a well-known diagnostic marker of ongoing infection [21], [29], [30], and one important factor in the development of Ag85B-TB10.4 was to reserve ESAT-6 as a diagnostic/infection marker. We therefore examined the usage of ESAT-6 to predict protection/infection status when using a BCG booster vaccine strategy that did not contain ESAT-6. Moreover, we also examined the correlation between CD4 T cell responses against the vaccine antigens obtained by flow cytometry shown in figure 3 with the bacterial levels six weeks post infection shown in figure 4.

**Figure 5:**
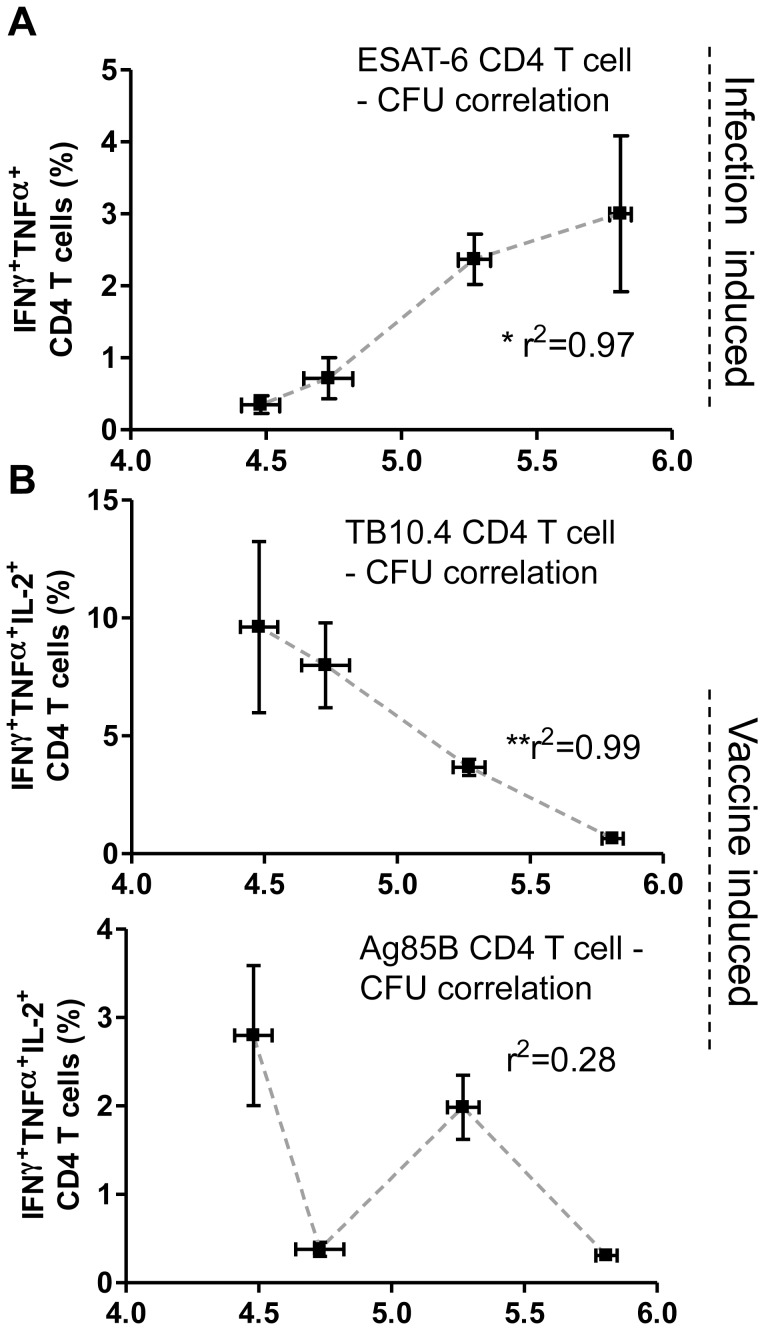
Correlates of protection six weeks after infection. A, the pulmonary responses obtained using ICS and flow cytometry six weeks after infection shown in figure 3 after stimulation with ESAT-6 (A) or the H4-vaccine antigens Ag85B- and TB10.4 (B) were correlated to the corresponding mean log_10_ CFU value shown in figure 4. Points represent mean percentage and SEM (vertical) of CD4 T cells producing IFNγ/TNFα (A) or IFNγ/TNFα/IL-2 (B) in response to stimulation with indicated antigens from 3 pools of two lungs plotted on the y-axis and mean and SEM (horizontal) log_10_ CFU values of mice lungs (N = 29 for non-vaccinated controls and n = 9−10 for the vaccination groups) on the x-axis. Each point represents one group in the order BCG/H4-IC31, BCG, H4-IC31, and non vaccinated from left. *, p<0.05, **, p<0.01, using Pearson’s product-moment correlation coefficient (r) and correlation test.

Our results showed that post infection, the response against ESAT-6 was highest in non-vaccinated mice which also had the highest levels of CFU (Fig. 3B, 5A and table S1). Thus, in all the vaccinated groups we observed a decreased ESAT-6 response after challenge, which however was most pronounced in the BCG/H4-IC31® group. By comparing the ESAT-6 response (specifically ESAT-6 specific IFNγ+TNFα+ T cells) with the corresponding CFU levels, a strong correlation (r^2^ = 0.97, p = 0.0179) was observed between the post-challenge ESAT-6 response and the outcome of disease/CFU levels. This correlation was not observed to the same degree with ESAT-6 specific IFNγ^+^TNFα^+^IL2^+^ T cells (r^2^ = 0.79, p = 0,11, data not shown). In contrast to ESAT-6 specific CD4 T cells, we observed a significant negative correlation between the IFNγ^+^TNFα^+^IL2^+^ responding CD4 T cells against TB10.4 and the bacterial levels (r^2^ = 0.99, p = 0.0029, figure 5B). However, there was no correlation between the IFNγ^+^TNFα^+^IL2^+^ Ag85B specific T cells in the lungs and the bacterial levels (r^2^ = 0.28, p = 0.4645; figure 5B). Finally, the CD8 T cell response against ESAT-6 was dominated by IFNγ^+^TNFα^+^ effector cells, and these cells also showed a significant positive correlation with the bacterial level (r^2^ = 0.99, p = 0.0085; figure S2). Moreover, the CD8 T cell response against TB10.4 and Ag85B (Fig. S2B) also correlated with the CFU levels (Fig. S2A).

In summary, the magnitude of the CD4 and CD8 response against ESAT-6 correlated significantly with bacterial burden and inversely with vaccine efficacy. In contrast, the TB10.4 CD4 response showed a positive correlation with vaccine efficacy.

## Discussion

### Boosting with H4-IC31® Induces Protective Polyfunctional Memory T Cells

In this study we demonstrated that boosting BCG with H4-IC31® or with H4/DDA/MPL both enhanced and prolonged the BCG-primed immune response (Fig. 1). This in turn led to a significant improvement in protection provided that the protection induced by BCG itself did not exceed 1.1 Log_10_ reduction in Lung CFU. This indicate that in the mouse model used in this study only protection of approximately 1.5 Log10 could be achieved. Although several studies have shown that BCG protection can be improved by a subunit or viral based booster vaccine (reviewed in [31]) few have identified the protective T cell phenotypes. The improved response in the BCG/H4-IC31® consisted of an increase in 1) the amounts of CD4 T cells expressing all three cytokines IFNγ, IL-2 and TNFα and 2) an increase in IL-2/TNFα-expressing cells (Fig. 2). These cell types have been suggested to represent effector memory- and central memory-like cells and have furthermore been associated with protection against intracellular pathogens such as M.tb and *Leishmania major* [25]. Our data also showed a correlation between the amounts of triple positive IL-2^+^TNFα^+^IFNγ^+^ vaccine-specific CD4 T cells induced by immunization and the subsequent level of protection against infection with M.tb (Fig. 2 and 4). In agreement with our results, Dey *et al.* also observed induction of IL-2^+^TNFα^+^IFNγ^+^ and IL-2^+^TNFα^+^ CD4 T cells after boosting BCG with a DNA-vaccine encoding α-crystallin (Rv2031c) [32], and boosting BCG with the MVA-vector expressing Ag85A also resulted in increased numbers of polyfunctional CD4 T cells with similar phenotypes as observed in the present study [33]. However, regarding the protective role of IL-2^+^TNFα^+^IFNγ^+^ T cells against M.tb infection there have been conflicting results [25], [33], [34], [35], [36]. An increase in Ag85A-specific polyfunctional CD4 T cells after boosting BCG with MVA85A did not improve protection [33]. Moreover, a recent study showed that humans with active TB displayed high numbers of IFNγ^+^IL-2^+^TNFα^+^ triple positive cells specific for M.tb-antigens, while latently infected individuals did not. Moreover, the level of triple positive CD4 T cells actually declined when TB-patients with active disease were treated [37]. This is in contrast to other studies which, in line with our results, showed a correlation between triple positive cells and protection [38]. One difference that might explain the conflicting observations may relate to the different antigens used in the different studies.

### Correlates of Disease Progression

We observed a significant correlation between protection and the induction of IFNγ^+^IL-2^+^TNFα^+^ triple positive polyfunctional TB10.4 specific CD4 T cells, indicating an important protective role for these cells. In contrast, the level of Ag85B-specific CD4 T cells did not correlate with protection measured 6 weeks after infection. Interestingly, recent studies have shown that Ag85B in particular is expressed primarily in the early stages of an infection with M.tb, i.e. within the first 2−3 weeks [27], [39]. Accordingly Ag85B immune responses would only be expected to correlate with bacterial numbers in the early stages of infection.

We have previously shown that Hybrid1 (Ag85B-ESAT-6) is highly immunogenic in humans and protective against TB in several animal models and that it could improve BCG conferred protection [40], [41]. We recently showed that exchanging ESAT-6 with TB10.4 resulted in an equally efficient protection against TB in mice [21]. One advantage of exchanging ESAT-6 with TB10.4 is that it reserves the diagnostic marker ESAT-6 for disease screening. The current study confirmed the ability of ESAT-6 to predict the infection level in mice as demonstrated previously (Fig. 5 and [21]). ESAT-6 responses also correlated with bacterial burden and pathology severity in cattle [29] and non-human primates [41]. Moreover, humans recently exposed to M.tb exhibiting a high ESAT-6 response were more likely to develop active TB compared to ESAT-6 low-responders [30], [42], [43].

Interestingly, we also observed a positive correlation between the bacterial burden and the level of CD8 T cell responses against ESAT-6 and both the vaccine antigens (Fig. S2). This suggests that CD8 responses could also be a useful tool for monitoring vaccine efficacy and disease progression.

In summary, our results extend the studies of Skeiky *et al*. who showed that H4-IC31® could boost BCG immunity leading to a reduction in CFU levels and slightly prolonged survival in guinea pigs [44]. In extension of these data we showed that the mechanism by which H4-IC31® boosted BCG-primed protective immunity in the mouse model was by inducing an increased and prolonged response consisting of CD4 T cells with an effector-memory and central-memory phenotype. Importantly, our vaccine strategy did not compromise ESAT-6 based diagnostics. H4-IC31® is presently in clinical trials as a BCG-booster vaccine.

## Materials and Methods

### Ethics Statement

Experiments were conducted in accordance with the regulations set forward by the Danish Ministry of Justice and animal protection committees by Danish Animal Experiments Inspectorate Permit 2009/561-1655 or 2004-561-868 and in compliance with European Community Directive 86/609 and the U.S. Association for Laboratory Animal Care recommendations for the care and use of laboratory animals. The experiments were approved by the SSI animal ethics board headed by DVM Kristin E. Engelhart Illigen.

### Animal Handling

Studies were performed with 6- to 8-week-old female F1 crossing of inbred male C57BL/6 and female Balb/c mice from Harlan Scandinavia (CB6F1). This F1 mouse stain was used due to broad recognition of vaccine antigens compared to the parent strains. Mice were housed in appropriate animal facilities at Statens Serum Institut and infected animals housed in cages contained within laminar flow safety enclosures (Scantainer from Scanbur, Denmark) in a separate biosafety level 3 facility at Statens Serum Institut. All mice were fed radiation sterilized 2016 Global Rodent Maintenance diet (Harlan, Scandinavia) and water ad libitum. All animals were allowed a one week rest prior to initiation of the experiments.

### Bacteria

*M.tb* Erdman was grown at 37°C on Middlebrook 7H11 (BD Pharmingen) agar or in suspension in Sauton medium (BD Pharmingen) enriched with 0.5% sodium pyruvate, 0.5% glucose, and 0.2% Tween 80. BCG Danish strain 1331 was grown at 37°C in Middlebrook 7H9 medium (BD Pharmingen). All bacteria were grown to log phase and then stored at −80°C in growth medium at ∼5×10^8^ CFU/ml. Bacteria were thawed, placed in an ultrasound-bath for five minutes, clumps dispersed by forcing bacteria through a syringe, washed and diluted in PBS prior to infection.

### Antigens

Recombinant H4 (Ag85B-TB10.4) and Ag85B were produced in *E. coli* and purified using column-purification as previously described [21]. For restimulation of cell cultures with TB10.4, peptide-mixtures with 18-mer peptides covering the antigen-sequence with an overlap of 10 amino acids per peptide were used at a final concentration of 2 µg/ml per single peptide in the mixture [23]. For Ag85B and ESAT-6 restimulation, recombinant Ag85B produced in *E. coli* was used. Cell cultures were all stimulated with media only and ESAT-6 as a negative control antigen before infections, as well as concanavalin A to assess cell viability.

### Immunizations and Experiment Protocols

Mice immunized with BCG received a single dose of 5×10^6^ CFU of BCG Danish 1331 per mouse injected s.c. in a volume of 0.2 ml at the base of the tail at week 0 of the experiment. Mice that received H4-IC31® were immunized two times subcutaneously (s.c.) on the back at the base of the tail with experimental vaccines containing 0.5 µg H4 formulated with the IC31® adjuvant in a total volume of 200 µl 19 and 22 weeks after BCG immunizations. The adjuvant IC31®consists of a mixture of the peptide KLK (NH2-KLKL5KLK-COOH) and the oligodeoxynucleotide ODN1a (oligo-(dIdC)13) provided by Intercell. Doses were 100 nmol peptide and 4 nmol oligonucleotide. Control mice immunized with only H4-IC31®, without prior BCG-vaccination, received H4-IC31® simultaneously with the BCG/H4-IC31® boosted group (see figure 1A). Mice were challenged with M.tb seven weeks after the last H4-IC31® booster (29 weeks post BCG). Regarding the three experiments shown in figure S1, in Experiment 1, mice were primed with BCG as described above and boosted 34 and 36 weeks later with H4 emulsified in dimethyl dioctadecyl ammonium bromide (DDA; 250 µg/dose; Eastman Kodak) and monophosphoryl lipid A (MPL; 25 µg/dose; Avanti Polar Lipids) in a volume of 0.2 ml, as described previously [21]. In this experiment, mice were challenged with M.tb five weeks after the final booster vaccination. Bacterial levels were measured six weeks after infection. The second experiment shown in figure S1 (Exp. 2) is the same experiment as shown in figure 4, and the third experiment (Exp. 3) is an exact replicate of experiment two (where mice were challenged seven weeks after the final H4-IC31 booster and protection assessed six weeks later).

### Experimental Infections and Enumeration of CFU to Assess Protection

Mice challenged by the aerosol route were infected with ∼100 CFU of *M.tb* Erdman/mouse with an inhalation exposure system (Glas-Col) as previously described [21]. These mice were killed 6 weeks after challenge. Numbers of bacteria in the spleen and lung were determined by serial 3-fold dilutions of individual whole-organ homogenates on 7H11 medium. Organs from the BCG-vaccinated animals were grown on medium supplemented with 2 µg of 2-thiophene-carboxylic acid hydrazide/ml to selectively inhibit the growth of any residual BCG bacteria in the test organs. Colonies were counted after 2–3 weeks of incubation at 37°C. Protective efficacies were expressed as log_10_ bacterial counts in immunized mice compared with bacterial counts in the controls.

### Lymphocyte Cultures

PBMCs were purified on a density gradient using lympholyte® for mammals (Cedarlane, Canada) and splenocyte and lung lymphocyte cultures were obtained by passage of organs through a 100-µm nylon cell strainer (BD Pharmingen). After washing, cells were cultured in roundbottom microtiter wells (96-well plates; Nunc) containing 2×10^5^ cells in a volume of 200 µl of c-RPMI (RPMI 1640 supplemented with 5×10^−5^ M 2-ME, 1% (v/v) premixed penicillin-streptomycin solution (Invitrogen Life Technologies), 1 mM glutamine, and 10% (v/v) FCS). Based on previous dose-response investigations, the mycobacterial antigens were all used at 2 µg/ml, whereas Concanavalin A was used at a concentration of 1 µg/ml as a positive control for cell viability. Supernatants from triplicate cultures were harvested from cultures after 72 h of incubation for the investigation of IFNγ by sandwich ELISA. For intracellular cytokine analysis by flow cytometry, 1−2×10^6^ cells from blood, spleen or lung were cultured in c-RPMI and stained as described below.

### Cytokine ELISA

A sandwich ELISA was used to determine the concentration of IFNγ in culture supernatants, as described previously [24].

### Flowcytometric Analysis

Intracellular cytokine staining of T cells was done as described previously [24]. Briefly, cells were stimulated for 1 hour with 2 µg/ml antigen and subsequently incubated for 5 hours with 10 µg/ml brefeldin A (Sigma-Aldrich) at 37°C and then stained for surface markers, permeabilized using the BD Biosciences cytoperm/cytofix kit according to the manufacturer’s instructions and subsequently stained for intracellular cytokine expression. Samples were run on a FACS Canto six-colour flow cytometer from BD Biosciences. All antibodies were purchased from BD Biosciences except anti-IFNγ, which was purchased from eBioscience. In the results section, the percentage of total responding CD4/CD8 T cells were determined by summing the percentage of cells producing IFNγ, TNFα or IL-2. The relative proportions of cells producing different combinations were calculated by Pestle and SPICE software and represent the proportions of subpopulations relative to total responding CD4 or CD8 T cells. Background responses from media controls were subtracted.

### Statistical Analysis

For comparisons of pulmonary responses measured after infection by flow cytometry, individual subpopulations of T cells were compared to non-vaccinated mice using 1-way ANOVA and Bonferroni-correction for multiple comparisons (Fig. 3). For multiple comparisons of CFUs, one-way ANOVA was used with Newman-Keuls post-test for multiple comparisons and statistical differences marked by asterisks in figures and explained in figure legends. For statistical analysis of log_10_ protection individual student’s t-test was made for each experiment. Correlations between mean log_10_ CFUs and responses from ICS flow cytometry were performed using Pearson’s product-moment correlation coefficient (r) and correlation test. The coefficient of determination (r^2^) shows the amount of variation shared by the two variables. Graphpad prism 5.0 software was used for analysis.

## Supporting Information

Figure S1 **Boosting with H4 improves suboptimal BCG-derived protection.** In three separate experiments the level of protection observed from BCG/H4 booster groups was comparable. However, H4-boosting only significantly improved BCG-derived protection when protection from BCG itself was below 1.1 log_10_ CFU in the lungs. In experiment number one BCG was boosted with H4-DDA/MPL. In experiment two and three boosting was performed with H4-IC31® (see Materials and Methods for details). Bars represent individual log_10_ CFU values deducted with the mean value of the non-vaccinated control group from the same experiment. *, p<0.05 analysed by student’s t-test comparing log_10_ reduction in CFU in the lungs for each experiment separately.
(TIF)

Figure S2 **CD8 T cell response correlate with CFU levels six weeks after infection.** A, the pulmonary responses obtained using ICS and flow cytometry six weeks after infection shown in figure 3 after stimulation with Ag85B, TB10.4 or ESAT-6 were correlated to the corresponding mean log_10_ CFU value shown in figure 4. Points represent mean percentage and SEM (vertical) of CD8 T cells producing IFNγ in response to stimulation with indicated antigens from 3 pools of two lungs plotted on the y-axis and mean and SEM (horizontal) log_10_ CFU values of mice lungs (N = 29 for non-vaccinated controls and n = 9−10 for the vaccination groups) on the x-axis. Each point represents one group in the order BCG/H4-IC31, BCG, H4-IC31, and non vaccinated from left. *, p<0.05, **, p<0.01, using Pearson’s product-moment correlation coefficient (r) and correlation test. B, pulmonary CD8 T cell responses six weeks after infection. Lung lymphocytes from three pools consisting of two half lungs per pool were used from each group for intracellular cytokine analysis by flow cytometry. Cells were stimulated with the antigens specified in the graph. Bars represent the proportions of lung CD8 T cell subsets producing different cytokines in response to stimulation as indicated on the X-axis. Background levels obtained in media-stimulated samples has been deducted. *, p<0.05, **, p<0.01, ***, p<0.001, proportion of cytokine producing CD8 T cells subsets compared to non-vaccinated controls using one-way ANOVA and Tukey’s post-test for multiple comparisons.
(TIF)

Table S1 **Percentages of lung T cells producing cytokines in the different groups as well as the corresponding log_10_ CFU values with SD and SEM.** Log_10_ CFU values with SD and SEM are shown for each group, as well as the percentage of lung CD4 T cells producing IFN-γ, TNFα and IL-2 simultaneously after stimulation with the indicated antigens analysed by intracellular cytokine staining and flow cytometry. * Three pools consisting of two half lungs per pool were used from each group for stimulation.
(XLSX)
