# Supplementary figures and images for: The HyVac4 Subunit Vaccine Efficiently Boosts BCG-Primed Anti-Mycobacterial Protective Immunity
Source: PLoS One. 2012 Jun 29;7(6):e39909. doi: 10.1371/journal.pone.0039909 (PMC3386939; doi:10.1371/journal.pone.0039909)

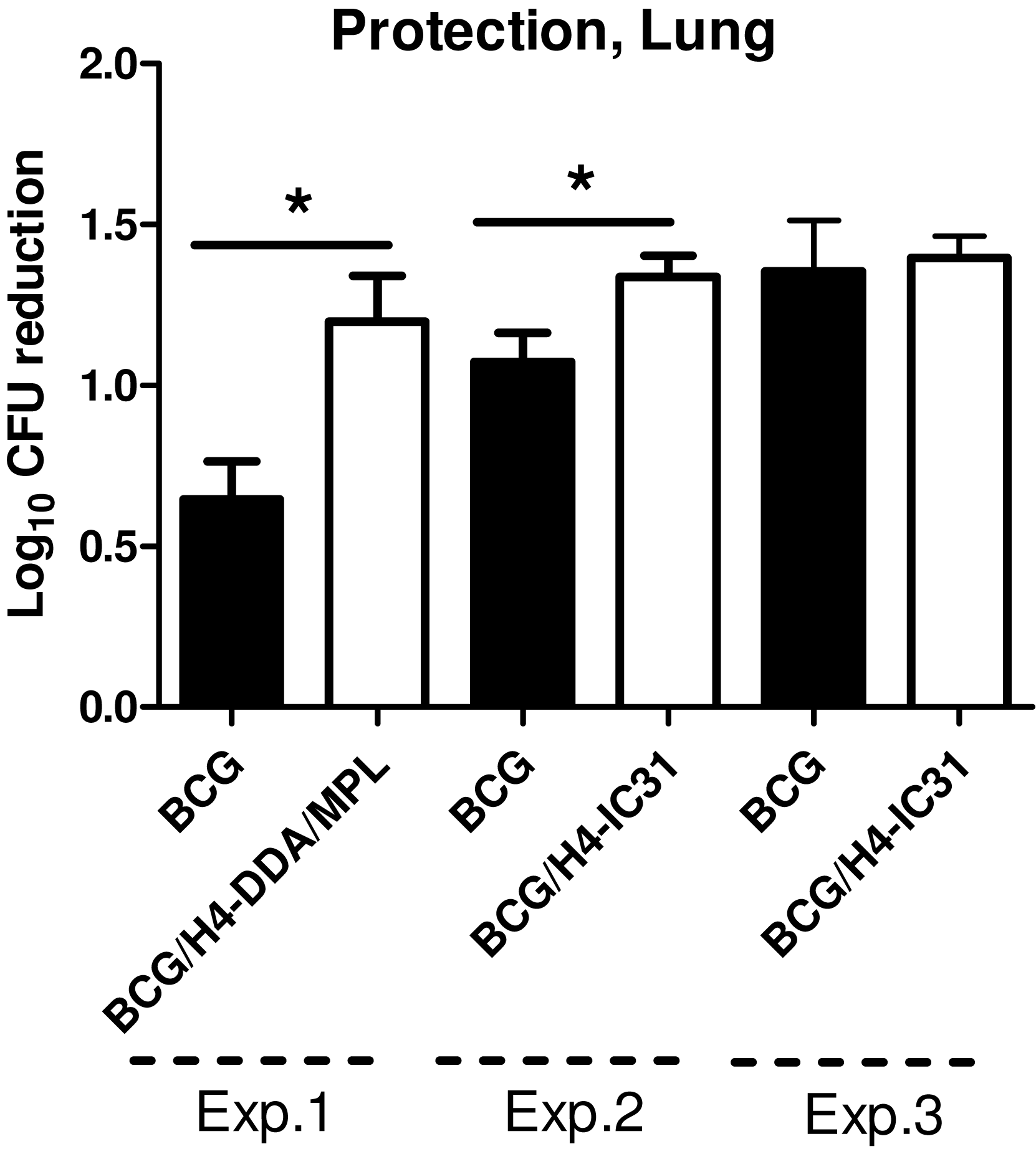

Supplement: Figure S1 — Boosting with H4 improves suboptimal BCG-derived protection. In three separate experiments the level of protection observed from BCG/H4 booster groups was comparable. However, H4-boosting only significantly improved BCG-derived protection when protection from BCG itself was below 1.1 log10 CFU in the lungs. In experiment number one BCG was boosted with H4-DDA/MPL. In experiment two and three boosting was performed with H4-IC31® (see Materials and Methods for details). Bars represent individual log10 CFU values deducted with the mean value of the non-vaccinated control group from the same experiment. *, p<0.05 analysed by student’s t-test comparing log10 reduction in CFU in the lungs for each experiment separately. (TIF) [file pone.0039909.s001.tif]

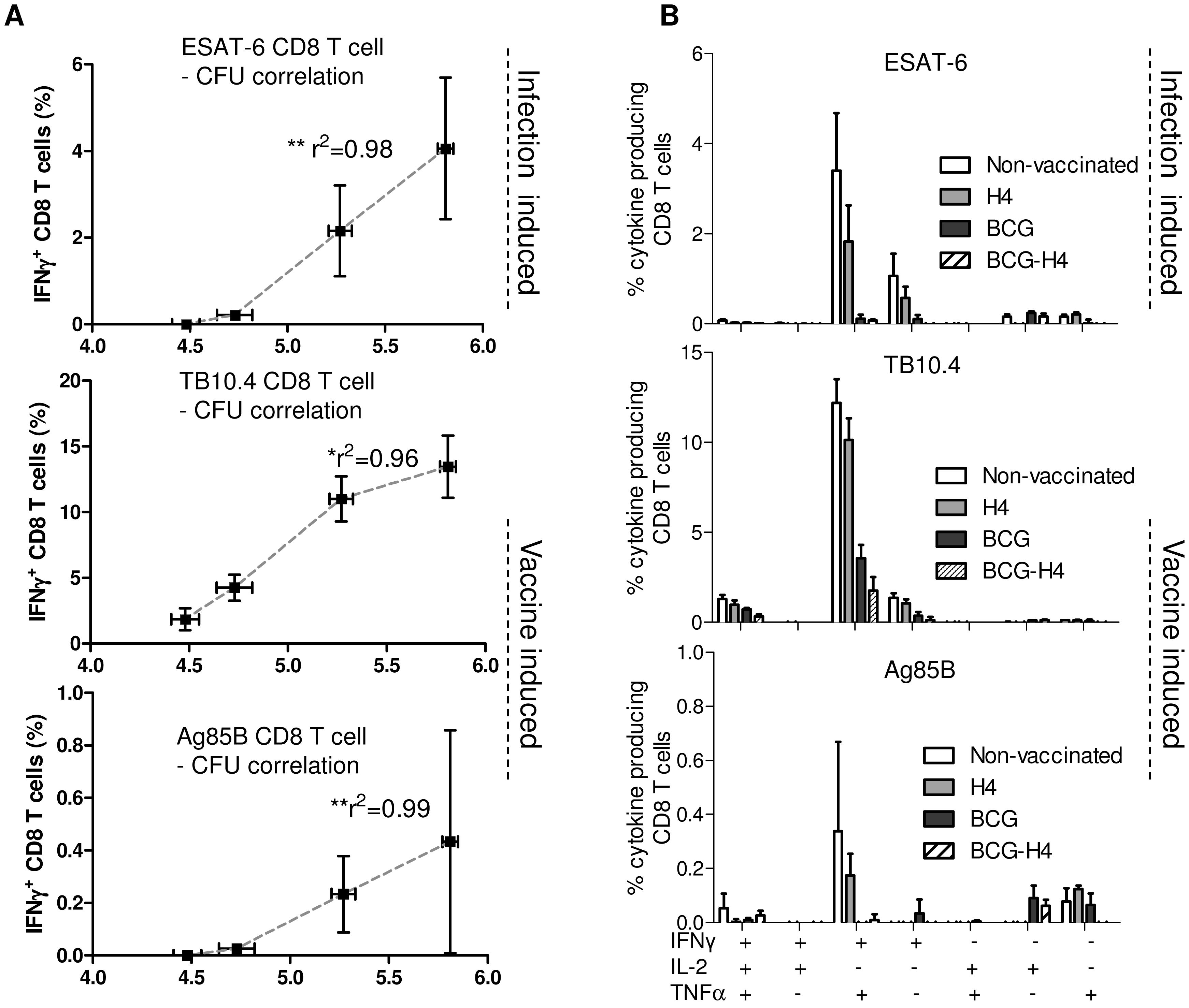

Supplement: Figure S2 — CD8 T cell response correlate with CFU levels six weeks after infection. A, the pulmonary responses obtained using ICS and flow cytometry six weeks after infection shown in figure 3 after stimulation with Ag85B, TB10.4 or ESAT-6 were correlated to the corresponding mean log10 CFU value shown in figure 4. Points represent mean percentage and SEM (vertical) of CD8 T cells producing IFNγ in response to stimulation with indicated antigens from 3 pools of two lungs plotted on the y-axis and mean and SEM (horizontal) log10 CFU values of mice lungs (N = 29 for non-vaccinated controls and n = 9−10 for the vaccination groups) on the x-axis. Each point represents one group in the order BCG/H4-IC31, BCG, H4-IC31, and non vaccinated from left. *, p<0.05, **, p<0.01, using Pearson’s product-moment correlation coefficient (r) and correlation test. B, pulmonary CD8 T cell responses six weeks after infection. Lung lymphocytes from three pools consisting of two half lungs per pool were used from each group for intracellular cytokine analysis by flow cytometry. Cells were stimulated with the antigens specified in the graph. Bars represent the proportions of lung CD8 T cell subsets producing different cytokines in response to stimulation as indicated on the X-axis. Background levels obtained in media-stimulated samples has been deducted. *, p<0.05, **, p<0.01, ***, p<0.001, proportion of cytokine producing CD8 T cells subsets compared to non-vaccinated controls using one-way ANOVA and Tukey’s post-test for multiple comparisons. (TIF) [file pone.0039909.s002.tif]
